# Supplementary material for: Conserved Imprinted Genes between Intra-Subspecies and Inter-Subspecies Are Involved in Energy Metabolism and Seed Development in Rice
Source: Int J Mol Sci. 2020 Dec 17;21(24):9618. doi: 10.3390/ijms21249618 (PMC7765902; doi:10.3390/ijms21249618)
Supplement: Supplementary file 1 [file ijms-21-09618-s001.zip › Supplementary Data/Supplement Figures.doc]

**Figure S1.** Identification of hybrids with InDel markers. (**A**) Identification of hybrids between 9311 and Nip with Indel marker RID2-1. The samples in lanes 25 and 26 are 9311 and Nip, and the rest are potential hybrids. (**B**) Identification of hybrids between 9311 and ZS97 with Indel marker RID9-21. The samples in lanes 25 and 26 are 9311 and ZS97, and the rest are potential hybrids. (**C**) Identification of hybrids between ZH11 and Nip with Indel marker RID6-9N. The samples in lanes 25 and 26 are Nip and ZH11, and the rest are potential hybrids.

**Figures S2.** Scatter diagram of the Nip ASRs in comparison with the 9311 ASRs in reciprocal hybrids of 9311 and Nip. (**A**) Embryo replicates 1 of 9N. (**B**) Endosperm replicates 1 of 9N. (**C**) Embryo replicates 2 of 9N. (**D**) Endosperm replicates 2 of 9N. (**E**) Embryo of N9. (**F**) Endosperm of N9.

**Figures S3.** Scatter diagram of the ZS97 ASRs compared with the 9311 ASRs in reciprocal hybrids of ZS97 and 9311. (**A**) Embryo of 9ZF1. (**B**) Endosperm of 9Z. (**C**) Embryo of Z9. (**D**) Endosperm of Z9.

**Figures S4.** Scatter diagram of the ZH11 ASRs compared with the Nip ASRs in reciprocal hybrids of ZH11 and Nip. (**A**) Embryo of ZN. (**B**) Endosperm of ZN. (**C**) Embryo of NZ. (**D**) Endosperm of NZ.

**Figure S5.** Expression levels of imprinted genes between endosperm and embryo. (**A**) The expression levels of MEGs in the embryo (yellow) and endosperm (green) of R9Z. (**B**) The expression levels PEGs in the embryo (yellow) and endosperm (green) of R9Z. (**C**) The expression levels of MEGs in the embryo (yellow) and endosperm (green) of RZN. (**D**) The expression levels of PEGs in the embryo (yellow) and endosperm (green) of RZN. (**E**) The expression levels of MEGs in the embryo (yellow) and endosperm (green) of R9N. (**F**) The expression levels of PEGs in the embryo (yellow) and endosperm (green) of R9N. (**G**) The heatmap of imprinted genes expression in R9N. (**H**) The heatmap of imprinted genes expression in R9Z. (**I**) The heatmap of imprinted genes expression in RZN.

**Figure S6.** The validation of 6 imprinted genes between two different reciprocal crosses. (**A**) Os02g0626400 and Os11g0455500 between R9N and R9Z. (**B**) Os06g0498150 and Os06g0538900 between R9N and R9Z. (**C**) Os07g0596600 and Os11g0283500 between R9Z and RZN.

**Figure S7.** The frequency distribution of the numbers of shared imprinted genes based on 10000 simulation tests among three sets of reciprocal crosses and between any two sets of reciprocal crosses. (**A**) The simulation of frequency distribution of the number of shared imprinted genes in all three combination. (**B**) The simulation of frequency distribution of the number of shared imprinted genes between R9N and R9Z. (**C**) The simulation of frequency distribution of the number of shared imprinted genes between R9N and R9Z. (**D**) The simulation of frequency distribution of the number of shared imprinted genes between R9Z and RZN. X-axis indicated the numbers of shared imprinted genes. Y-axis indicated the times of 10000 simulation tests corresponding to the number of shared imprinted genes.

**Figure S8.** The frequency distribution of the numbers of shared imprinted genes based on 10000 simulation tests between this study and the previous work. The X-axis indicated the numbers of shared imprinted genes between two studies, while the Y-axis indicated the times of 10000 simulation tests corresponding to the number of shared imprinted genes.

**Figure S9.** Distribution of MEGs in the subcellular location.

**Figure S10.** Distribution of PEGs in subcellular location.

**Figure S11.** Carbon fixation pathway. Red box, MEGs involved in the pathway. The name of MEGs was labeled in red font and placed above the red box. Green box, the enzymes involved in starch and sucrose metabolism existed in rice. White box, the enzymes existed in other species.

**Figure S12.** Phenylpropanoid biosynthesis pathway. Red box, MEGs involved in the pathway. The name of MEGs was labeled in red font and placed above the red box. Green box, the enzymes involved in starch and sucrose metabolism existed in rice. White box, the enzymes existed in other species.

**Figure S13.** Plant hormone signal transduction. Red box, MEGs involved in the pathway. The name of MEGs was labeled in red font and placed above the red box. Green box, the enzymes involved in starch and sucrose metabolism existed in rice. White box, the enzymes existed in other species.
